# Utility of Magnetic Resonance Arthrography in the Age of Expanded Magnetic Resonance Imaging Capabilities: A Global Survey Perspective

**DOI:** 10.7759/cureus.71526

**Published:** 2024-10-15

**Authors:** Danika Baskar, Girish Gandikota, Rajesh Botchu, Dharmendra K Singh, Rafeh Khan, Vijay Papineni

**Affiliations:** 1 Radiology, University of North Carolina School of Medicine, Chapel Hill, USA; 2 Musculoskeletal Radiology, The Royal Orthopaedic Hospital, Birmingham, GBR; 3 Radiology, Vardhman Mahavir Medical College and Safdarjung Hospital, New Delhi, IND; 4 Radiology, Sheikh Shakhbout Medical City, Abu Dhabi, ARE

**Keywords:** contrast, global survey, joint, magnetic resonance arthrography, magnetic resonance imaging, musculoskeletal, radiology

## Abstract

Background

Magnetic resonance imaging (MRI) arthrography has been a mainstay in the diagnosis of musculoskeletal (MSK) joint-related pathology for decades. With the advent of MRI and further advancements incorporating powerful magnetic fields, the radiological landscape is undergoing a shift. The objective of this study is to evaluate the relevance and preference for MRI arthrography versus MRI among MSK radiologists in our current clinical practice across a range of geographic locations.

Methods

A global survey of 52 MSK radiologists was conducted to understand current practices, preferences, and recommendations related to the adoption of MRI arthrography and advanced MRI technologies. Responses were analyzed using descriptive statistics, and significant trends were compiled to present the overall preferences of the participants from their clinical experience.

Results

The majority of the respondents were from the UK (65%), followed by India (13%), the United States (11.5%), and the United Arab Emirates (8%). Of the radiologists surveyed, 98% currently perform MRI arthrogram procedures within their practice, with 27% of clinicians conducting more than 100 MRI arthrogram procedures annually. Regarding the adoption of 3T MRI scanners, 79% of respondents affirmed they have access to the technology within their respective institutions. Concerning the choice of contrast agents used, 84% preferred dilute gadolinium, 8% used saline, 4% used both gadolinium and saline, and another 4% opted for alternative agents. Despite the growing accessibility and utility of 3T MRI, MRI arthrograms remain a favored procedure, especially for shoulder pathology and postoperative cases. For the hip and wrist, the preference leans toward 3T MRI, except for more complex diagnostic purposes or postoperative cases.

Conclusions

The study’s findings suggest that while the advent of the 3T MRI has impacted the frequency with which MRI arthrograms are conducted, it cannot entirely replace the precision and specificity MRI arthrography offers for particular joint pathologies. As institutions expand upon their MRI and imaging infrastructure, further research in this area may help guide MSK radiologists in selecting the most appropriate imaging modality for patients on a case-by-case basis.

## Introduction

Ever since the introduction of the technology in the 1970s, magnetic resonance imaging (MRI) has revolutionized the evaluation of musculoskeletal (MSK) disease as it allows for noninvasive and high-resolution imaging of the anatomy of interest without the use of ionizing radiation [1-3]. The increased sensitivity of MRI compared to traditional radiography and improved specificity from bone scans make it optimal for a variety of diagnostic purposes to detect structural changes in MSK and soft tissue structures [2,4]. Several technical advances have greatly expanded MRI capabilities, with some notable features including higher field strengths using specialized coils, increased variety of pulse sequences, and improvements in post-processing techniques [3]. The availability of remarkable imaging software and hardware additionally allows for enhanced visualization of structural anatomy. While most MSK MRI was typically acquired using an intermediate field strength of around 1.5T, the integration of the 3.0T MRI in clinical settings has significantly increased. Even more powerful systems like the 7.0T MRI have been approved for patient care integration as of 2017 and continue to drive forward clinical and research advancement within the field of MSK radiology [3,5]. As the development of higher-field imaging systems continues to improve parameters like signal-to-noise ratio, tissue contrast, and image resolution, there are several technical considerations that must be accounted for to ensure the capture of high-quality images for clinical assessment. Challenges related to imaging artifacts, cartilage mapping, and postoperative or hardware imaging have been noted, which may deem certain studies inconclusive [3]. This may then require the patient to undergo reexamination or further investigation through other potentially more invasive means.

MRI arthrography is an alternative to MRI and is especially valuable for the evaluation of joint-related disorders. This technique utilizes a dilute contrast agent that is injected into the joint being investigated under direct fluoroscopic guidance [3]. The introduction of the contrast agent allows for distention of the joint compartment and allows for improved visualization of articular surfaces and surrounding structures. In comparison to MRI, MRI arthrography offers improved diagnostic accuracy for the detection of various ligamentous and cartilaginous pathologies of the shoulder, hip, and wrist, such as rotator cuff injuries, superior labrum anterior-posterior (SLAP) lesions of the shoulder, femoroacetabular impingement, acetabular labral tears, and triangular fibrocartilage (TFCC) tears, to name a few [3,6-8]. The enhancement that results from the use of the intra-articular contrast injection can be beneficial in identifying certain anatomical anomalies and physiological variants [7]. It should be noted that while MRI arthrography offers its own unique advantages, it is an invasive procedure that involves radiation, takes additional time to complete when compared to undergoing isolated MRI, and presents the risk of contrast-related hypersensitivity effects in the patient.

Prior studies evaluating the diagnostic abilities of MRI in comparison to MRI arthrography have demonstrated mixed results with regard to particular joint-related pathology. Within the scope of shoulder evaluation, significantly increased sensitivity of MRI arthrography has been noted in detecting SLAP tears, partial-thickness supraspinatus tears at the articular surface, tears of the anterior labrum, and in postoperative shoulder pathology when compared to conventional 3.0T MRI [9-11]. However, studies still continue to emphasize the importance of MRI examination as the first-choice imaging modality for diagnosing rotator cuff tears and labral lesions [12,13]. Literature focused on imaging of the hip joint has demonstrated nearly equivalent ability between 3.0T MRI and MRI arthrography in identifying acetabular labral tears, while other studies have found increased sensitivity of MRI arthrography over 3.0T MRI in detecting chondral defects of the acetabulum [14-17]. With regard to the wrist joint, studies have found MRI arthrography to be more sensitive and specific in visualizing ligament tears and lesions of the TFCC, which were undetectable on MRI [18,19]. Although MRI arthrography has been suggested to replace MRI and even diagnostic arthroscopy in identifying etiologies of chronic wrist pain, the possibility of false-positive findings due to micro-perforations in ligamentous structures is a concern that has been identified in the literature [19,20]. In addition to looking specifically at practices derived from evidence for common pathologies of interest, understanding a broader view of MRI versus MRI arthrography recommendations within clinical settings across a wide geographic distribution can provide insight into the current atmosphere of examination preferences.

As MRI technology continues to evolve and expand our diagnostic abilities, it is important to investigate whether MRI arthrography provides significant utility or benefit and under what specific clinical circumstances this may hold true. An international survey completed by the European Society of Musculoskeletal Radiology (ESSR) in 2018 explored the use of MRI arthrography within clinical practice among its members and found that the proportion of MRI arthrography performed was higher at orthopedic hospitals compared to general hospitals [21]. This data provides excellent initial insight into practices within European institutions and a basis for expanding efforts to evaluate how changing technology and the availability of advanced MRI capabilities influence clinical practices on a broader global scale. This study aims to learn about MRI and MRI arthrography utilization for the investigation of MSK pathology through an expanded international survey-based approach. Knowledge learned from insight into current clinical practices may reveal opportunities to highlight existing challenges and identify areas of future study.

This article was previously presented at The Royal College of Radiologists (RCR) Global Congress Scientific Meeting on October 1, 2022.

## Materials and methods

Determination of human subject research

This project was submitted for human subjects research determination. This submission was determined to not constitute human subjects research and did not require institutional review board approval. No individual patient medical data was reviewed as part of the analysis.

Sample size determination and recruitment

A global survey of 52 MSK radiologists was conducted to learn more about the use of MRI arthrography and advanced MRI technologies. Study participants were recruited from MSK radiology practices and professional societies across a range of geographic areas. The determination of the sample size for this survey was influenced by the specialized nature of MSK radiology. Due to advancements in noninvasive imaging techniques that offer enhanced image quality and anatomical detail, those who are trained in the practice of MRI arthrography and continue to implement this study regularly in their diagnostic methods have declined over time. The further subspecialization of radiologists within the MSK discipline also limits the pool of potential survey participants, and those who regularly perform MRI arthrography among this cohort represent a niche area of expertise. Given these challenges, efforts to maximize response rates included extending the survey period time and sending multiple reminders to professional groups, institutions, and individuals to recruit as many participants as possible on a global scale. Only MSK radiologists who were trained in MRI arthrography techniques and continue to perform this invasive diagnostic test were included in the study. Those who were not trained in MRI arthrography or did not perform arthrograms in their practice were excluded from study participation. A comprehensive literature review was also conducted to supplement the study efforts.

Study questionnaire

The study team designed a questionnaire to understand current practices and recommendations surrounding MRI arthrography and MRI examination in relation to their clinical utility for specific joint-related pathology. The study questionnaire also incorporated key areas of discussion highlighted in the existing relevant literature (Table 1). Open textboxes were included in the questionnaire to allow respondents to provide relevant commentary, clarify or justify their responses, and share insights from their clinical practice. The survey was distributed to all participants electronically through an online survey platform. Reminders were sent periodically over the three-month study period to individuals and through professional forums and societies to gather responses distributed across a range of geographic regions.

**Table 1:** Summary of literature reviewed CTA, computed tomography angiography; FAI, femoroacetabular impingement; MR, magnetic resonance; MRI, magnetic resonance imaging; SLAP, superior labrum anterior posterior

Author	Joint	Methodology	Conclusions
Magee et al. (2004) [9]	Shoulder	MRI and MRI arthrograms of 20 professional baseball players with shoulder pain vs. a control group of 50 nonprofessional athletes	MRI arthrography is more sensitive for detecting partial-thickness supraspinatus tears and labral tears than conventional MRI. High-performance athletes are likely to benefit from MRI arthrograms.
Magee (2009) [10]	Shoulder	150 shoulder MRI and MRI arthrograms reviewed retrospectively from patients who underwent arthroscopy	3-T MRI arthrography showed increased sensitivity for the detection of partial-thickness articular surface supraspinatus tears, anterior labral tears, and SLAP tears compared with conventional MRI.
Liu et al. (2019) [12]	Shoulder	Systematic review and meta-analysis of 14 studies involving 1,216 patients	MRI arthrography had the highest sensitivity and specificity for detecting labral lesions compared to MRI and CTA, suggested primarily for chronic shoulder symptoms.
Liu et al. (2020) [13]	Shoulder	Meta-analysis of 12 studies involving 1,030 patients and 1,032 shoulders	MRI is recommended as a first-choice imaging modality for the detection of rotator cuff tears, although MRI arthrography has higher sensitivity and specificity.
Smith et al. (2011) [14]	Hip	Meta-analysis of 19 papers assessing 881 hips	3.0-T MRI is at least equivalent to 1.5-T MRI arthrography in detecting acetabular labrum tears and possibly superior in detecting cartilage defects.
Chopra et al. 2018 [16]	Hip	68 patients with clinical FAI underwent both 1.5T MRI arthrograms and 3T MRI, with subsequent hip arthroscopy	3.0-T MRI is recommended over MRI arthrography for detecting acetabular labral tears due to its noninvasive, fast, and convenient methodology.
Magee (2015) [17]	Hip	43 consecutive patients had both conventional hip MR and MRI arthrograms, followed by arthroscopy	MRI arthrography and 3.0-T MRI are near equivalent in the diagnosis of acetabular labral tears; MRI arthrography is more sensitive for detecting chondral defects.
Pahwa et al. (2014) [18]	Wrist	MRI and MRI arthrograms performed in 53 patients with wrist pain, compared with operative findings	MRI arthrography is more sensitive and specific for the evaluation of wrist ligament tears and TFCC lesions, recommended over MRI for chronic wrist pain.
Omar et al. (2019) [19]	Wrist	37 patients with chronic wrist pain examined by MRI and MRI arthrograms, with arthroscopic correlation in 25	MRI arthrography provides higher diagnostic confidence and more accurate delineation of wrist ligament injuries than conventional MRI.

Data collection/statistical analysis

Respondents were asked to complete the electronic study questionnaire by selecting the single best response for each question based on their collective expertise and implemented methods from clinical practice. Survey responses were compiled once the study questionnaire completion period was closed, and results were analyzed using descriptive statistics. Trends in responses were compiled to present overall preferences and potential areas for further investigation. Comments from open textboxes were also analyzed for recurring responses that were significant to justifying preferences for MRI versus MRI arthrography.

## Results

**Figure 1:**
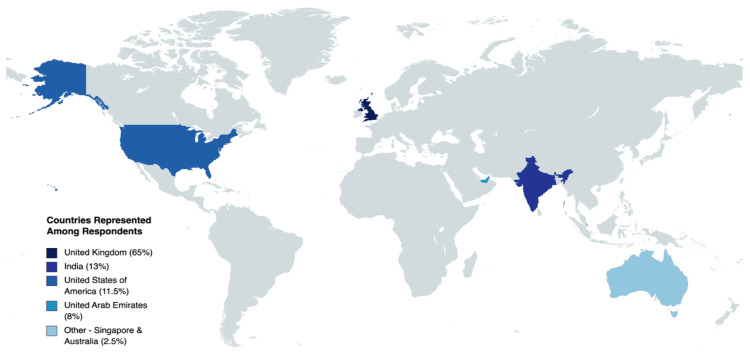
Countries represented by survey respondents (n = 52)

**Figure 2:**
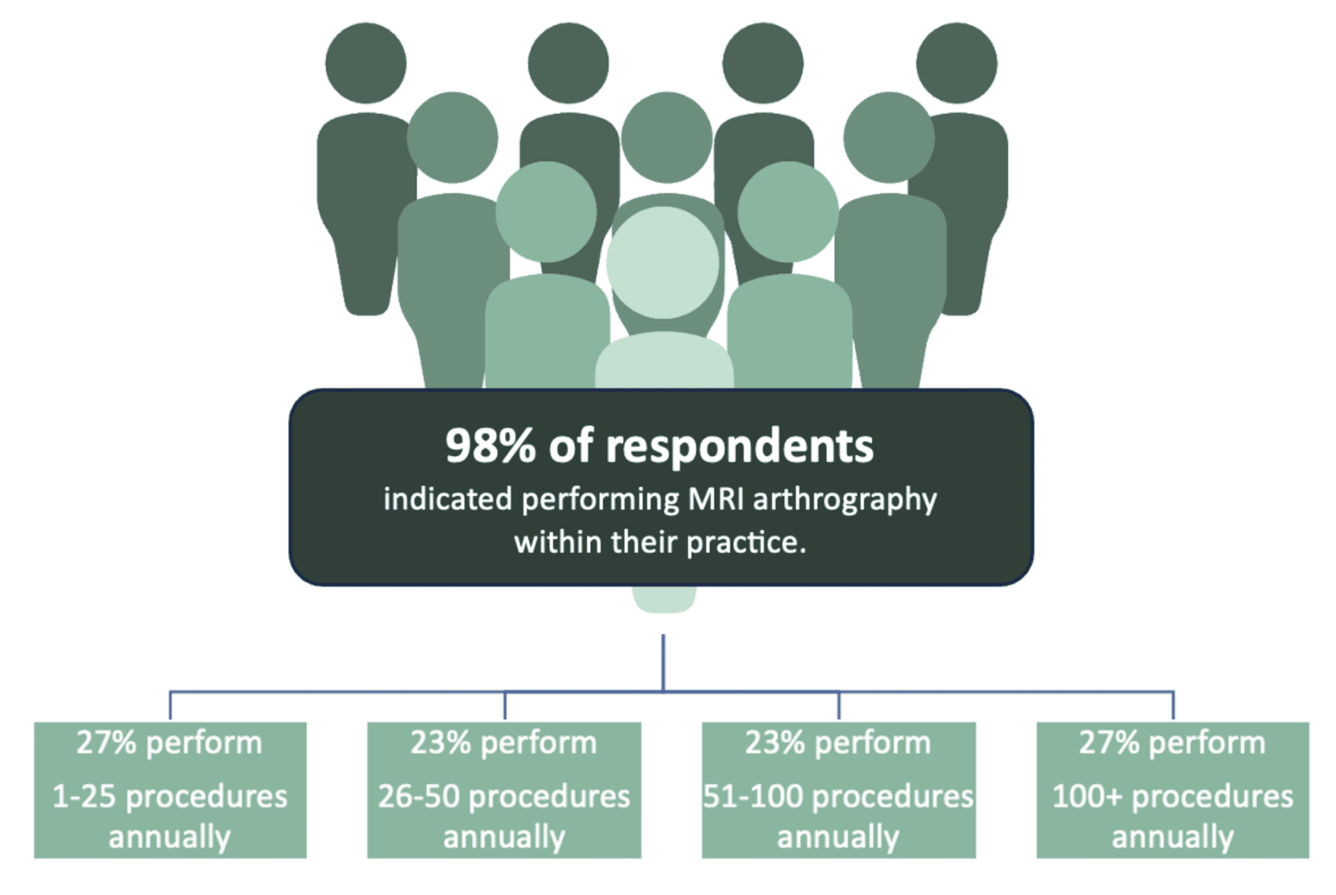
MRI arthrography procedures performed annually across respondents (n = 52)

A total of 52 responses were analyzed from MSK radiologists who participated in the study. Key findings from the analysis of received responses are summarized in Table 2. Participants indicated encountering a broad range of MSK pathologies in their practice and continue to perform both diagnostic and therapeutic procedures, including MRI arthrography. The patients for which respondents were performing MRI arthrography were primarily adult patients with chronic joint issues impacting their quality of life. The majority of respondents were from the United Kingdom (65%), followed by India (13%), the United States (11.5%), the United Arab Emirates (8%), and other parts of the world, including Singapore and Australia (2.5%) (Figure 1). Ninety-eight percent of survey respondents indicated they currently perform MRI arthrography procedures within their clinical practice. Out of the respondents who use MRI arthrogram procedures in their practice: 27% perform over 100 procedures, 23% perform between 51 and 100 procedures, 23% perform 26-50 procedures, and 27% perform 1-25 procedures on an annual basis (Figure 2).

**Table 2:** Summary of critical findings from the survey (n = 52) MRI, magnetic resonance imaging; MSK, musculoskeletal; SLAP, superior labrum anterior posterior; TFCC, triangular fibrocartilage

Survey question	Results
Current practice of MRI arthrography	98% of MSK radiologists perform MRI arthrography in their current practice.
Access to 3T MRI scanners	Yes: 79%; No: 21%
Contrast agents used in MRI arthrography	Dilute gadolinium: 84%; saline: 8%; both: 4%; other: 4%
Perceived need for MRI arthrography with the advent of 3T MRI	56% of respondents feel there is less need for MRI arthrography, while 44% believe MRI arthrography is still necessary.
Factors influencing preference for MRI arthrography over MRI	Predominantly patient-specific clinical questions, followed by the surgeon’s preference and additional diagnostic workup after MRI
Most common indications for MRI arthrography of the shoulder	Labral tears, SLAP lesions, and shoulder instability
Most common indications for MRI arthrography of the hip	Acetabular labral tears, primarily for complex or postoperative cases
Most common indications for MRI arthrography of the wrist	TFCC and ligamentous injuries, especially in complex or postoperative cases

With regard to access to a 3T MRI scanner, 79% of participating MSK radiologists noted having this technology available within their respective institutions, while 21% noted not having access to equipment with this magnetic field strength. Regarding preference for contrast agent of choice when performing MRI arthrography, 84% of respondents indicated using dilute gadolinium, 8% used only saline, 4% used both gadolinium and saline, and 4% used an alternative agent (IV gadolinium).

Considering the advent of 3T MRI, the majority of survey respondents (56%) agreed there is less of a need for performing MRI arthrograms, while 44% were in disagreement with this statement supporting the need for MRI arthrography along with MRI in today’s practice. In regard to factors that dictate a preference for MRI arthrography over conventional 3T MRI examination, the majority agreed that this decision was primarily driven by patient-specific clinical questions. This response was followed by a tie between the deciding factors for MRI arthrography over MRI being the ordering surgeon’s preference and requiring additional diagnostic workup after obtaining an MRI exam. Respondents also noted other factors to be important in preference for MRI arthrography compared to MRI, such as waiting time to obtain appointment or exam availability, consideration for the specific joint or pathology in question, and potential postoperative pathology such as labral tears.

**Figure 3:**
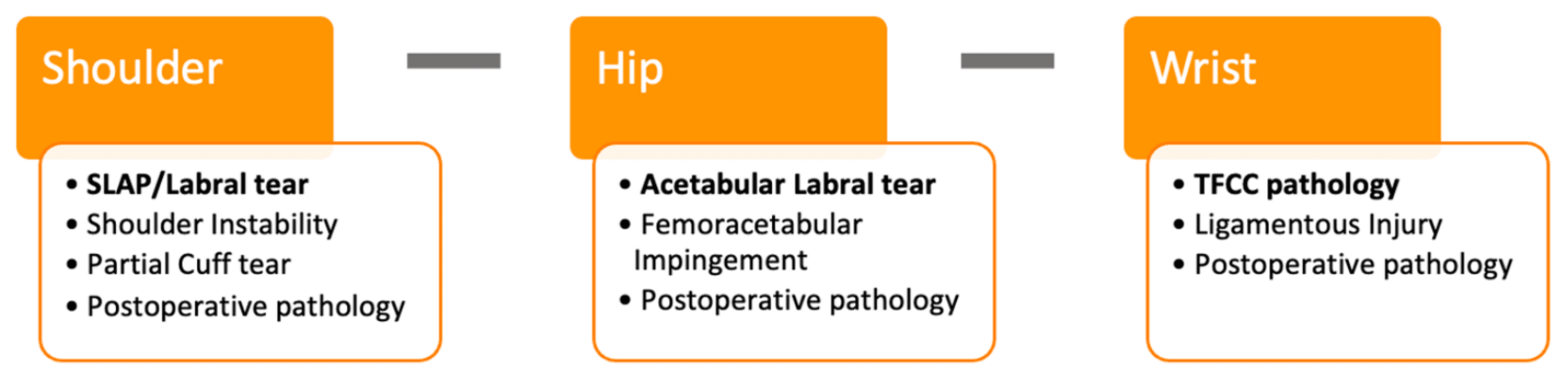
Most common indications for which respondents recommend MRI arthrography of specific joints The most common pathology for which MRI arthrography of a specific joint was recommended is in bold font. MRI, magnetic resonance imaging; SLAP, superior labrum anterior posterior; TFCC, triangular fibrocartilage Image credit: Danika Baskar

Evaluation of pathology related to commonly investigated joints was also surveyed with attention to the shoulder, hip, and wrist, for which the results are summarized in Figure 3. Labral tears and SLAP lesions were the most common indications for MRI arthrography evaluation of the shoulder, followed by an assessment of shoulder instability. For the hip joint, acetabular labral tears were found to be the most common clinical hypotheses for which MRI arthrography is preferred. TFCC and ligamentous injury evaluation were together agreed to be diagnoses for which MRI arthrography would be strongly recommended. Comments obtained from survey respondents with regard to MRI arthrography examination of the shoulder, hip, and wrist reveal that MRI arthrogram examination can be especially useful for MSK radiologists and is sought out to answer complex clinical questions requiring further investigation within the postoperative setting.

## Discussion

MRI technology has greatly impacted the ability to visualize MSK pathology and guide appropriate care [2-4]. MRI arthrography has been a longstanding tool in investigating various joint-related pathologies and can be especially helpful in complex or postoperative patient cases [3,8,10]. Critical differences between MRI and MRI arthrography revolve around the invasive nature of arthrography, potential effects of contrast in a patient, time in conducting the complete examination, and visual enhancement of anatomy provided by each method. As the capabilities of imaging technology continue to expand, it is important to understand the utility of our current diagnostic methods and how they evolve over time.

Studies within the existing literature focused on the use of higher field strength 3.0T MRI compared to MRI arthrography evaluation present mixed results with respect to the evaluation of the shoulder, hip, and wrist [12-14,19,20]. Despite the incorporation of both methods in MSK radiology practice, little is known from the perspective of practicing clinicians about how opting for MRI and MRI arthrography has changed with improved technology, whether one exam is preferred over the other, and how certain clinical questions influence decisions to recommend a specific modality. Our study aimed to investigate these areas through a global survey of MSK radiologists to obtain further insight into these decisions within the current clinical practice landscape. A similar study completed in 2018 surveying members of the ESSR found that with the exclusion of the spine, approximately one out of every 20 MSK MR examinations is an MRI arthrography, with a slightly higher proportion being performed in orthopedic hospitals [21]. This study also reported that the shoulder is the most frequently investigated joint using MRI arthrography, and both instability and labral injury were the two most common indications for choosing this examination [21]. Among our survey respondents who provided insight into practices across a global perspective, 98% of MSK radiologists indicated they currently perform MRI arthrography procedures, with 27% estimating over 100 procedures completed every year. 3T MRI capabilities were present in most institutions at which respondents practiced; however, 21% noted not having access to higher-field strength technology. Consistent with the survey completed by ESSR members, dilute gadolinium was the contrast agent of choice and was used by the majority of respondents for MRI arthrography examinations. While there was slightly higher agreement among our surveyed group that there is less of a need for performing MRI arthrography considering the capabilities of the 3T MRI, this statement was almost fairly split as 56% of participants agreed with this statement and 44% disagreed. Most respondents agreed that patient-specific clinical factors are the primary driver dictating preference for MRI arthrography over conventional 3T MRI investigation of a joint in question.

Considering the preference for MRI arthrography evaluation of the shoulder joint among survey respondents in our study, labral tears and SLAP lesions were the most common indications, followed by shoulder instability. Acetabular labral tears and both TFCC as well as ligamentous injuries of the wrist were pathologies for which those surveyed preferred MRI arthrography of the hip and wrist, respectively. Overall, it was also agreed that arthrography can be a valuable tool for the clinical evaluation of complex cases and in the postoperative setting. These findings, especially with respect to MRI arthrography investigation of the shoulder joint, were consistent with the previous study completed by members of the ESSR, as well as literature comparing MRI and MRI arthrography evaluation of similar injuries [11,20,21]. While evidence for MRI versus MRI arthrography preferences of the hip joint is mixed, studies focused on the wrist joint note similarities between visualization of ligamentous injury or TFCC tears through MRI arthrography and participant preferences of our study [14,15,18,19].

Considering the extent of 3.0T MRI incorporation today, our study demonstrates that MSK radiologists are performing significantly fewer MRI arthrography exams in their clinical practice. While this finding is similar to those shared in existing literature, it is important to note that advanced MRI capabilities, specifically 3.0T MRI, cannot replace MRI arthrography as a diagnostic tool for MSK pathology. There is a strong predilection for recommending MRI arthrography for shoulder-related pathology, especially when postoperative concerns are in clinical question. While 3.0T MRI is preferred for evaluation of the hip and wrist joints, challenging cases and complex scenarios involving postsurgical care can lead MSK radiologists to choose MRI arthrography examination. A factor that also becomes limiting in the decision to perform a specific exam over the others is the availability of medical imaging technology and access to resources. Because of this, MRI arthrography becomes a more preferred examination in areas with limited advanced MRI capabilities to assist in the diagnosis of clinical concerns that may be readily addressed using 3.0T MRI at other institutions.

While this study provides an expanded and current perspective on MRI and MRI arthrography preferences among MSK radiologists across a wide geographical distribution, there are several limitations that must be highlighted. Given the challenges in recruiting MSK radiologists with specific expertise in MRI arthrography, a technique that has declined in use over time with the advent of powerful noninvasive imaging technologies, we acknowledge that a larger response sample would provide improved insight into trends that can inform clinical practices across a wider scope. Also, as the majority of study participants who completed the survey were from the UK, this may limit the generalizability of our findings. Inclusion of more varied clinical practice sites and learning more about the types of cases and patients served at each respondent’s institution may present opportunities to understand how these factors impact clinician recommendations. Interactions with other providers like orthopedic and sports medicine physicians might also have an effect on the types of examinations opted for by MSK radiologists. Additionally, resource availability and institutional capabilities can significantly change how examinations are performed. While these limitations pose challenges to gaining a deep understanding of MRI versus MRI arthrography examination preferences, our study is able to provide a cross-sectional presentation of current trends, drawing from a broad international perspective from which further study may be informed. Areas of future research may include investigating how orthopedic and subspecialty care representation and resource limitation influence decisions toward MR practices. Learning about changes in practice preference over time can also be incredibly informative in mapping trends.

## Conclusions

MRI capabilities continue to expand our diagnostic abilities through technology that allows for improved visualization of pathology through noninvasive means. The findings of our study support that while the advent of the 3.0T MRI has impacted the frequency with which MRI arthrography exams are conducted, MSK radiologists agree that at this time it cannot entirely replace the precision and specificity MRI arthrography offers for particular joint pathologies of the shoulder, hip, and wrist. MRI arthrography offers a distinct advantage in helping to address clinical questions in the postoperative setting, complex clinical cases, and where previous MRI examinations may have been performed with indeterminate results. As institutions expand their imaging infrastructure and diagnostic capabilities to meet increasing demands for advanced modalities and diagnostic capabilities, further research in this area may help guide MSK radiologists in selecting the most appropriate imaging modality for their patients on a case-by-case basis.

## Appendices

MR Arthrogram vs. 3T MRI - Study questionnaire

The aim of this survey is to know how the evolution of better image resolution of 3T MRI was changed your current practice of doing MR arthrograms (MRA). It is aimed at MSK radiologists who are trained in interventional procedures. Please provide additional comments as necessary to support your selected responses.

Q1: Which part of the world do you practice radiology?

• UK

• USA/Canada

• Europe

• Middle East

• India

• Far East

• Other (please specify)

Q2: Do you perform MRA in your current practice?

• Yes

• No

Q3: How many MRA do you perform in a year?

• 1-25

• 26- 50

• 51- 100

• >100

Q4: When performing MRA, do you use:

• Dilute gadolinium preparation

• Normal saline

• Both the above

• Other (please specify)

Q5: Do you have access to 3T MRI scanner at your institute?

• Yes

• No

Q6: With the advent of 3T MRI, do you feel there is less need for doing an arthrogram (MRA)?

• Yes

• No

Q7: What factors dictate performing MRA vs using conventional MRI?

• Clinical question

• Surgeon’s preference

• Problem solving following plain MRI

• Waiting time/Availability

• Other(please specify)

Q8: For which shoulder pathology do you prefer MRA?

• SLAP/Labral tears

• To assess shoulder dislocation/instability

• Partial cuff tear

• All the above

• None of the above

• Other (specify)

Q9: For which of the hip pathology do you prefer MRA?

• Labral tear

• FAI

• All the above

• None of the above

• Other (please specify)

Q10: For which of the wrist pathology do you prefer MRA?

• TFCC pathology

• Ligament injury

• All the above

• None of the above

• Other (please specify)
